# Hexavalent chromium sequestration from electronic waste by biomass of *Aspergillus carbonarius*

**DOI:** 10.1080/21655979.2020.1780828

**Published:** 2020-06-24

**Authors:** Suresh Lakshmi, Kalidoss Suvedha, Ramesh Sruthi, Jayaprakash Lavanya, Sunita Varjani, Ekambaram Nakkeeran

**Affiliations:** a Department of Biotechnology, Sri Venkateswara College of Engineering, Sriperumbudur, India; b Paryavaran Bhavan, Gujarat Pollution Control Board, Gandhinagar, India

**Keywords:** Biosorption, Aspergillus carbonarius, biomass, e-waste, hexavalent chromium, Freundlich isotherm

## Abstract

The idea of eliminating noxious metal ions from electronic waste contaminated water has led to the use of the metal adsorbing ability of biological matter. The principle of an ion exchanger of biological origin is the key in exhibiting this metal binding feature of microbial biomass. In this study, dead biomass of *Aspergillus carbonarius* was immobilized using sodium alginate and tested as a biosorbent for hexavalent chromium elimination from effluent. Size and functional groups were characterized for the immobilized bead containing biomass. Optimization of boundary variables like bead size, biosorbent dosage, contact time, pH, and temperature were performed. Maximum elimination of 92.43% hexavalent chromium was achieved at pH 2.0 for 12 h at 37°C, with 20 g/25 mL adsorbent dosage. On application of adsorption isotherms, the data were found to fit Freundlich isotherm and exhibited a high value of correlation coefficient proving the ability of *A. carbonarius* biomass to act as an effective quencher of hexavalent chromium from electronic waste contaminated water.

## Graphical Abstract

**Figure:**
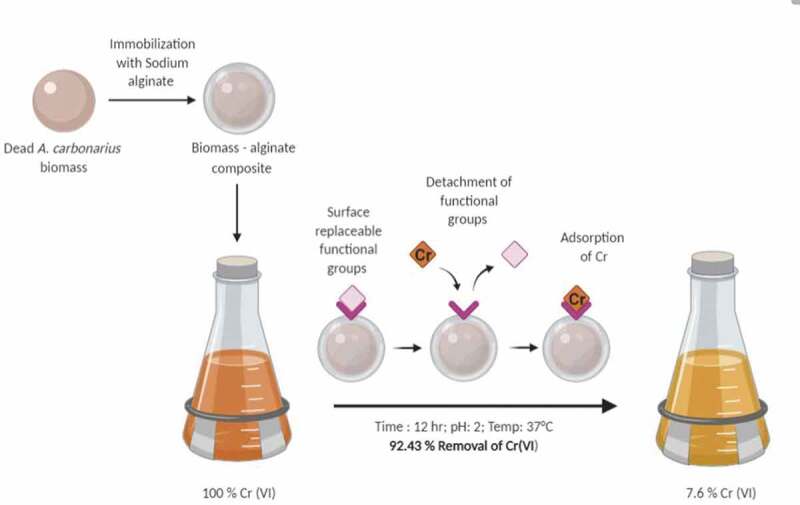

## 1. Introduction

Effluent discharge from industries is the primary source of heavy metals in waterbodies. Their toxic effect along with persistency and tendency to accumulate over time makes them a great hazard to human life and environment [1]. Electronic waste comprises any discarded electronic devices or unwanted by-products produced during the manufacturing of electronic products. According to a worldwide survey, it has been evident that the generation of e-waste has been increasing at the rate of 2-million-ton metrics per year, resulting in 49.8-million-ton metrics in 2018 [2]. In today’s global industrial scenario, electronic wastes are found to occupy a greater portion of toxic waste in landfills. The disposal process of electronic waste is of great alarm and may lead to several environmental impacts [3]. One such concern is that surface and groundwater resources are contaminated by the release of improperly treated or untreated industrial effluents [4]. Generally, e-wastes contain harmful heavy metals like chromium, beryllium, cadmium, mercury, and lead. However, concentration levels of lead and chromium are usually found high for instance it exceeds 1000 ppm in circuit board of a cellular phone [5]. Chromium is adverse in its effects causing cancer, major respiratory, gastrointestinal, and neurological diseases [6]. In nature chromium causes the most damage as it precipitates out of the air as dust particles and being water-soluble it can more easily move through ecosystems causing greater damage [7]. Therefore, the removal of such metals has become an important cause of concern for both humans and ecosystem. However, traditional treatment methods like ion exchange, precipitation, extraction, and osmosis are proven efficient only at low heavy metal concentrations while having cost limitations. In this context, adsorption processes have an edge over other methods due to the complete removal of pollutants from even very dilute solutions. Activated carbon though being a widely used material for adsorption faces limitations due to its cost inefficiency [8]. Besides, with rising environmental awareness, the need for environmentally friendly and affordable treatment technologies of biological origin is increasing [1,9]. There are three principle advantages of using biosorption as an alternate treatment process; first, it can be performed in-situ at the contaminated site, second this technology is environmentally benign and third the process is cost-effective. These form the primary motive for the development of a scaled-up biosorption technology to treat Chromium pollution [10]. For the last three decades, biomass as a replacement for activated carbon for sorption of different types of pollutants, namely, heavy metals, organic compounds, and dyes have been broadly studied [11].

Metal adsorption using large-scale ion-exchange resins, activated charcoal, and oxides of metals are found to be cost-inefficient with numerous constraints compared to microbe-based adsorption techniques, especially those employing fungi [12]. Ideally, live or inactivated filamentous fungal biomass are being employed in heavy metal sequestration [13–15]. The metal binding capacity of inactivated fungal biomass may vary greatly in being more efficient, equally efficient or even inefficient compared to live fungal biomass [16]. The cell walls containing polysaccharides, lipids, and proteins with varying functional moieties such as amine, hydroxyl, carboxyl, and phosphates play a significant role in interacting with metal ions enabling adsorption [17].

Several studies on biosorption to remove hexavalent chromium by fungal biomass particularly *A. niger* was performed [18,19]. Al-Asheh and Duvnjak [20], tested the ability of *A. carbonarius* to uptake copper (II) and chromium (III). However, Cr (III) is not considered as toxic since it cannot enter cells in contrast to Cr (VI) which can readily enter cells through the anionic transport systems making it more harmful [21]. Hence, biomass of *A. carbonarius*, generated as a waste from enzyme and pigment production process, could be used as an appropriate alternative for the removal of hexavalent chromium. The current work attempts to study the kinetics of adsorption of hexavalent chromium by calcium alginate-fungal dead biomass composite of *A. carbonarius* and its concordance with adsorption isotherms for depicting chromium uptake. Composite formation using a polymer shields the biomass from physical stress conditions by creating a secure micro-environment [22]. Alginate is the most commonly used biomaterial to entrap eukaryotic as well as prokaryotic cells due to its mild gelling, biocompatible, and biodegradable properties. The dead *A. carbonarius* biomass used for the study was obtained from the remnants of enzyme and pigment production process [23,24] hence, utilizing the organism to its fullest potential.

## 2. Materials and methods

### 2.1. Materials

Sodium alginate, calcium chloride, potassium dichromate were procured from M/s. Sisco Research Laboratories, India. All reagents used in this study were of analytical grade purchased from reputed manufacturers in India. *A. carbonarius* biomass was cultivated using cornflour medium in submerged fermentation [23].

### 2.2. Drying and size uniformization of fungal biomass

*A. carbonarius* biomass cultivated using submerged fermentation was collected from the fermentor, filtered to remove excess solvent, followed by sterilization at 121°C for 15 min and stored at 4°C for further use. Biomass washing was performed repeatedly with double distilled water to remove excess solvent from the biomass and the biomass was desiccated using a hot air oven at 65°C for 12–16 h. The desiccated biomass was pulverized into a fine powder, screened through standard sieves of 0.3 mm, and used for composite preparation [25].

### 2.3. Preparation of alginate-fungal biomass composites

For the preparation of alginate-fungal biomass composites, 4 g of fungal biomass was mixed with 2% sodium alginate. This solution was introduced dropwise with constant pressure through clipped pipette tips (200 µL and 1000 µL) of pre-defined diameter into 1 M calcium chloride solution. The formed beads have been filtered and stored in double-distilled water for further experimental use [26].

### 2.4. Preparation of synthetic effluent

Potassium dichromate (K_2_Cr_2_O_7_) of 0.2835 g was dissolved in 100 mL de-ionized water to prepare a stock solution of 1000 ppm Cr (VI) and was used for further studies [19].

### 2.5. Optimization of biosorption parameters

The synthetic effluent was used to perform biosorption studies under batch mode. For this study, 250 mL Erlenmeyer flasks containing the desired quantity of synthetic effluent was agitated at 120 rpm in an orbital shaker. Parameters for biosorption like adsorbent dosage, contact time, pH, and temperature were optimized as given below.

Adsorbent load and contact time were optimized by varying the dosage of beads between 5 and 25 g and duration of 4–24 h in 25 mL synthetic effluent. With the optimum conditions of adsorbent dosage and time, the optimum temperature and pH were determined using 20 g of adsorbent in 25 mL synthetic effluent for duration of 12 h by adjusting the pH range between 2 and 9 and temperature between 25°C and 50°C. The residual chromium concentration after biosorption in each process was ascertained by the 1,5-diphenylcarbazide method with a UV-Vis spectrophotometer [27]. The below formulas were used for calculating percentage biosorption and quantification of hexavalent chromium biosorbed by the adsorbent, respectively:

(1) $$Cr(VI)removal(\%)=\frac{(Ci-Cf)100}{Ci}$$

(2) $$A=\frac{(Ci-Cf)V}{W}$$

In Which, *A* corresponds to amount of hexavalent chromium biosorbed in mg/g, *C_i_* and *C_f_* are the initial concentration and final concentration of hexavalent chromium in mg/L, V is the volume of the prepared synthetic effluent in mL and W is the weight of adsorbent in g [19]. All the experiments were performed in triplicates and the mean values are reported. Individual biosorption percentage values are denoted as *x *± 5% standard error. Error bars represent standard error of mean values in the 95% confidence level.

### 2.6. Regeneration of alginate-fungal biomass composites

After biosorption, the alginate-biomass composite beads were obtained through filtration and regenerated by soaking in distilled water and desorbing in 0.5 M HCl for 12 h each [14]. These regenerated beads were kept at 4°C and used for a successive adsorption and desorption cycle under optimized conditions.

### 2.7. Adsorption kinetic studies

In addition to the studies on percent elimination of Cr (VI), adsorption isotherms were used to determine the interactions between adsorbate and adsorbent and the quantity of synthetic effluent adsorbed as a function of pressure and concentration by keeping temperature as a constant.

Langmuir’s isotherm applies well for monolayer adsorption. The Langmuir equation shown below describes a correlation between the total number of active sites on the adsorbate surface (i.e. the magnitude of adsorption) and pressure.

(3) $$Ceqe=\frac{1}{qmax}KL+\frac{Ce}{qmax}$$

where C_e_ corresponds to equilibrium concentration of material undergoing adsorption in this case the synthetic effluent (mg/L), *q_e_* corresponds to amount of hexavalent chromium adsorbed for each gram of the alginate-fungal biomass composite bead at equilibrium (mg/g). q_max_ (mg/g) and *K_L_* (L/mg) represent Langmuir constants associated with biosorption efficiency of adsorbent and adsorption rate correspondingly. Values for q_max_ as well as *K_L_* had been determined using the slope and intercept of the Langmuir graph plotted for *C_e_* vs. *C_e_/q_e_* [28].

Freundlich isotherm gives the empirical relationship between surface molecular concentration of biosorbent and concentration of adsorbate in solution. This non-linear adsorption model takes into consideration the monolayer adsorption of molecules on a heterogeneous surface suggesting the occurrence of different functional groups upon sorbent surface with different adsorption energy. The Freundlich isotherm equation is linearly given as:

(4) $$lnQe=lnKf+\frac{1}{n}lnCe$$

Wherein *Q_e_* denotes adsorption of hexavalent chromium for each gram of adsorbent (mg/g), *C_e_* is the equilibrium concentration of liquid adsorbate (mg/L), *K_f_* corresponds to Freundlich constant representing biosorption efficiency of adsorbent (mg/g), *n* denotes empirical constant, representing extent of biosorption (L/mg). Slope (1/*n*) and intercept (*ln K_f_*) of the linear plot of Freundlich equation denote extent of biosorption and efficiency of biosorbent, correspondingly [29].

### 2.8. FTIR analysis

Characterization of alginate-fungal biomass composites before and after Cr (VI) adsorption was performed using FTIR (M/s. Bruker Optik GmbH, Germany). The sampling was performed by mixing 10 mg of alginate-fungal biomass composites with KBr pellets. The FTIR spectra were acquired within the spectral range from 4000 to 400 cm^−1^ at a spatial resolution of 4 cm^−1^ with 64 scans and averaged [30]. The results of the spectral analyses of before and after Cr (VI) adsorption were compared.

## 3. Results and discussion

The dead *A. carbonarius* biomass obtained from the remnants of enzyme and pigment production process had been utilized as a potential biological adsorbent for elimination of hexavalent chromium. This serves as an effective mode of using the remains post enzyme and pigment production ensuring its utility to the maximum. The cost benefits associated with this are also considerably significant making the process more feasible for industrial application. For scaling up of this method, ideally, optimization of the pilot-scale batch process, isolation, and recovery of remnants of biomass from production houses and practical adsorption process using industrial electronic waste contaminated water are essentials for consideration.

### 3.1. Fourier transform infrared spectroscopy analysis

**Figure 1.**
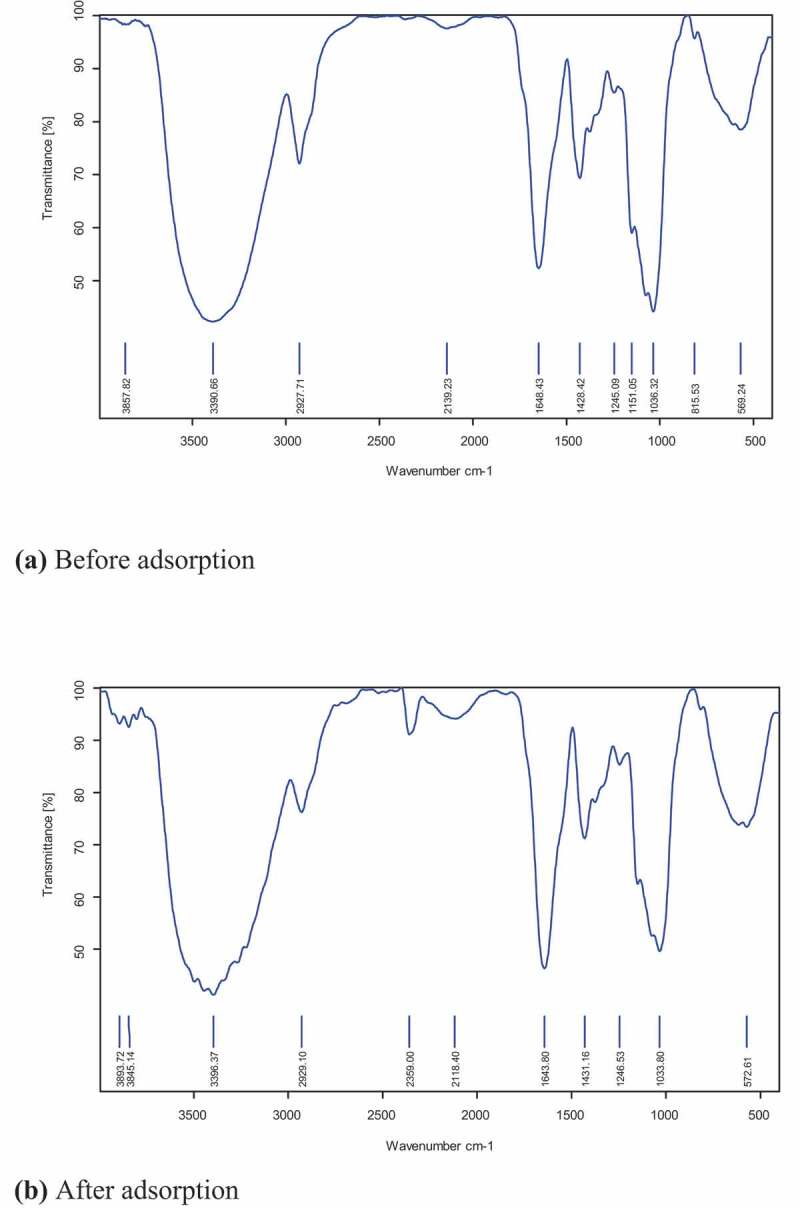
FTIR spectrum Cr (VI).

Figure 1(a,b) shows the FTIR analysis results before and after Cr (VI) adsorption individually. FTIR analysis of the polymer beads used in the study was performed primarily to identify which functional groups contributed to the adsorption of chromium and to strike a comparison between them before and after the adsorption process.

In the spectrum, each peak has been assigned a particular functional group. Lipid functionalities were characterized by the C = O mode of the side chain from the ester carbonyl group at 2927 cm^−1^ and a hydroxyl group at 3390 cm^−1^ (Figure 1(a)). However, shifts in the peaks at 1643 and 2929 cm^−1^ with the presence of additional aromatic functionality peaks between 2118 and 2359 cm^−1^ in Figure 1(b) distinguished the two plots and exhibited the characteristic of Cr (VI) adsorption.

### 3.2. Influence of bead size

**Figure 2.**
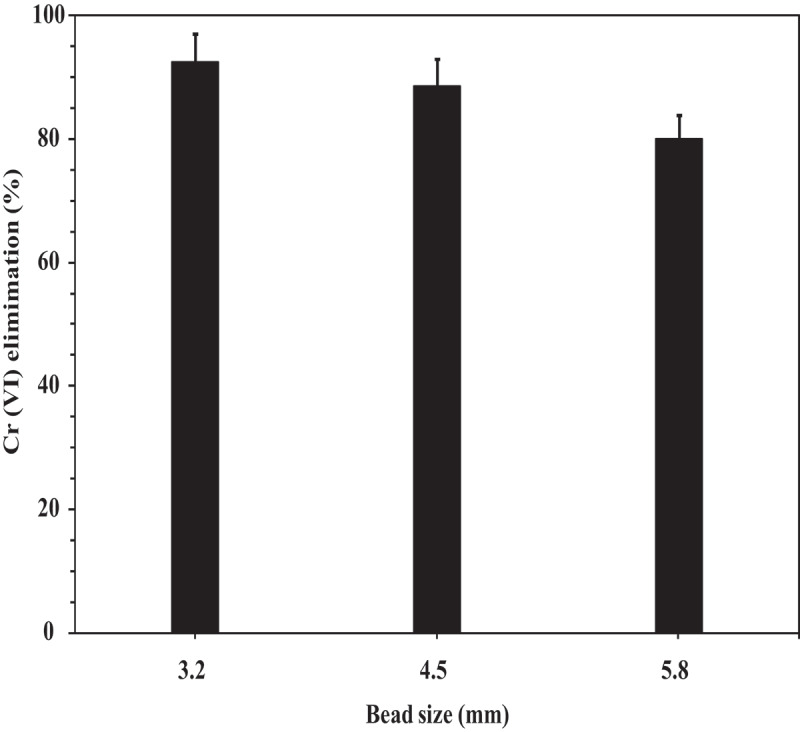
Influence of bead size on Cr (VI) elimination (Initial Cr (VI) concentration: 1000 ppm, Biosorbent dose: 20 g, pH: 2, Temperature: 37°C, Shaking speed: 120 rpm).

Interestingly, the biosorption efficiency and removal rate of Cr (VI) by alginate-biomass beads tended to increase gradually with decreasing bead size (Figure 2). This could be due to the increased surface area available for biosorption with reduced bead size. Also, with decreased bead size, diffusive mass-transfer limitations for hexavalent chromium solution is low when compared with higher bead sizes. Among the three compared bead sizes of 3.2, 4.5, and 5.8 mm at the same adsorption settings, maximum biosorption was observed for beads with 3.2 mm diameter. This is in accordance with the results published by [31].

### 3.3 Influence of pH

**Figure 3.**
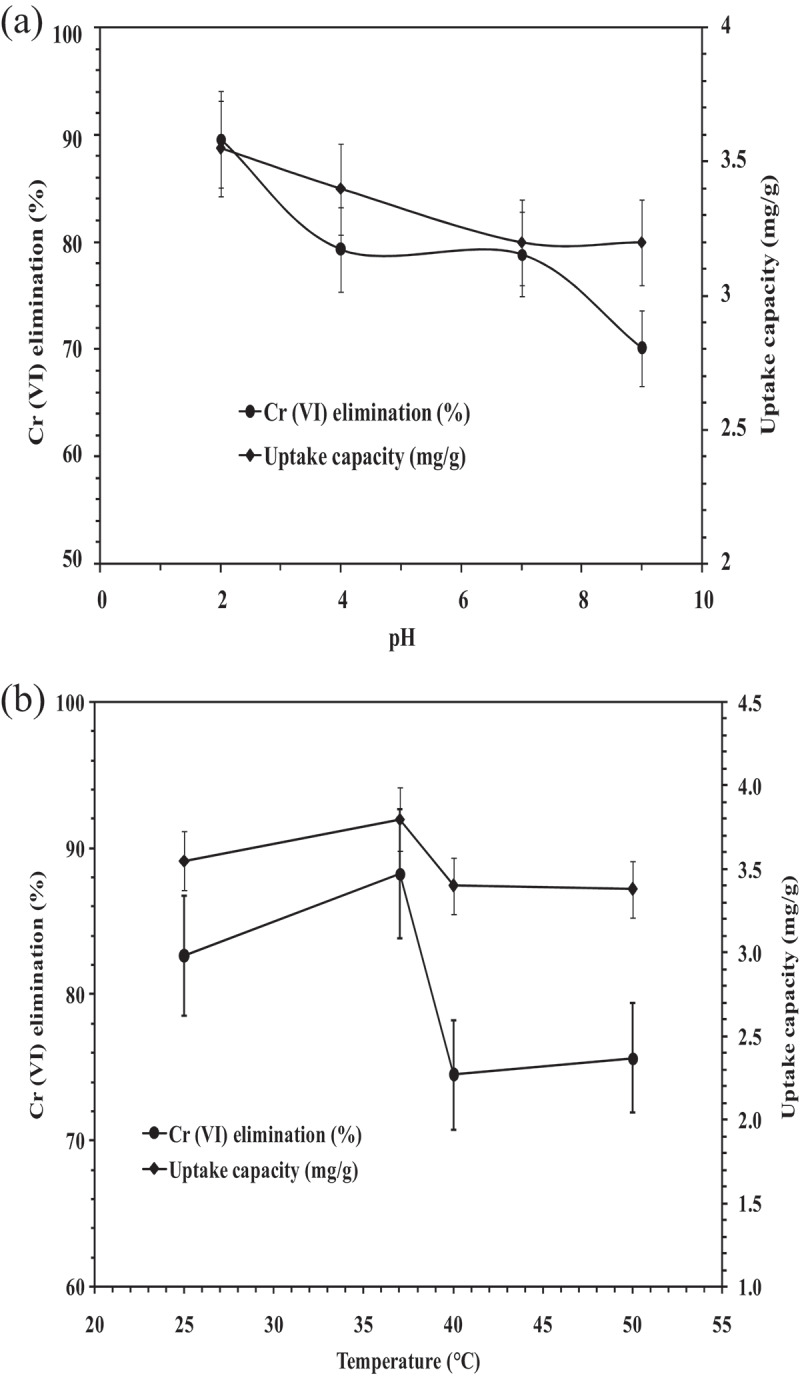
(a). Influence of pH on Cr (VI) elimination (Initial Cr (VI) concentration: 1000 ppm, Biosorbent dose: 20 g, Contact time: 12 h, Temperature: 37°C, Shaking speed: 120 rpm). Figure 3(b). Influence of temperature on Cr (VI) elimination (Initial Cr (VI) concentration: 1000 ppm, Biosorbent dosage: 20 g, Contact time: 12 h, pH: 2.0, Shaking speed: 120 rpm).

For adsorption in liquid phases, pH is a significant process parameter since it influences the surface charge of the adsorbent and metal chemistry in the solution. The pH dependence of adsorption is directly correlated with the competition between hydrogen ions for active sites on the adsorbent surface [32]. Figure 3(a) shows the elimination of hexavalent chromium by *A. carbonarius* biomass at various pH of the synthetic effluent. The initial chromium concentration of 1000 ppm, adsorbent dose of 20 g/25 mL and 12 h contact time were kept constant. The maximum Cr (VI) elimination of 89.64 ± 4.48% was obtained at pH 2. As the pH increased it resulted in a rise in the precipitation of insoluble and polymerized chromium oxide species CrO_4_^2-^ and HCrO^4-^ causing eventually leading to a decrease in the elimination of Cr (VI) [33]. Also, the increase in system’s pH resulted in protonation of active sites on surface of biomass. The Cr (VI) anions were not adsorbed at negatively charged sites because of electrostatic repulsion. Thus, higher adsorption capacity at low pH may be attributed to the presence of an increased amount of free H^+^ ions that can counteract the negatively charged biosorbent surface for effective diffusion of chromium ions. A similar result was also observed in a batch study using *Phanerochaete crysosporium* for adsorption of Cr(VI) [29].

### 3.4. Influence of temperature

Temperature is one of the most critical factors that determines the rate as well as the extent of biosorption for the given adsorbate. The effect of operating temperature on the adsorption of Cr (VI) is as shown in Figure 3(b). The experiment was performed at different temperatures of 25°C, 37°C, 40°C, and 50°C with a chromium stock solution of 1000 ppm concentration at pH 2.0 for 12 h. Increase in temperature improved the Cr (VI) elimination to a greater extent of 88.24 ± 4.41% at 37°C and thereafter decreased drastically (74.48 ± 3.72%) with increase in temperature of 40°C. This trend follows the results of [34]. These results suggested that the process is endothermic and follows Le Chatlier’s principle, i.e. it tends to attain equilibrium rapidly with an increase in temperature [35]. This creates large activation energy which ultimately led to higher adsorbate-adsorbent interaction. However, the increase in Cr (VI) elimination was observed until 37°C and thereafter decreased drastically with increasing temperature. This could be due to desorption of hexavalent chromium at elevated temperatures. Thus, the optimum temperature for adsorption was construed at 37°C.

### 3.5. Influence of biosorbent dosage

**Figure 4.**
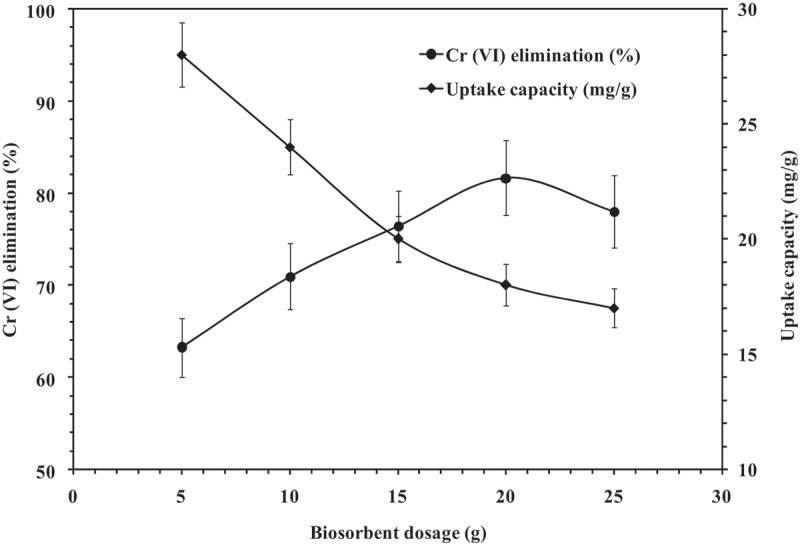
Influence of biosorbent dosage on Cr (VI) elimination (Initial Cr (VI) concentration: 1000 ppm, Contact time: 12 h, Temperature: 37°C, pH: 2, Shaking speed: 120 rpm).

Biosorbent dosage is another vital parameter influencing the cost of adsorbent dosage per unit volume of synthetic effluent in turn used to determine the feasibility of the process. Elimination of Cr (VI) by *A. carbonarius* as a function of biosorbent dosage is as shown in Figure 4. The study was performed with synthetic effluent of 1000 ppm Cr (VI) for 12 h at pH 2.0 and 37°C while varying the biosorbent dosage from 5 to 25 g of beads in 25 mL synthetic effluent. The optimum biosorbent dosage for Cr (VI) elimination was construed as 20 g/25 mL. At this dosage level, the percentage biosorption was found to be 81.67 ± 4.08%. As the biosorbent dosage increased, the Cr (VI) elimination capacity was found to increase from 63.23 ± 3.16% to 77.99 ± 3.89%. However, after a certain level increase in biosorbent dosage did not influence the biosorption capacity much. This may be because of the masking or aggregation of chromium ions on the adsorption sites of *A. carbonarius* that has resulted in the decrease of total surface area of the adsorbent that are accessible for further binding [36].

### 3.6. Influence of contact time

**Figure 5.**
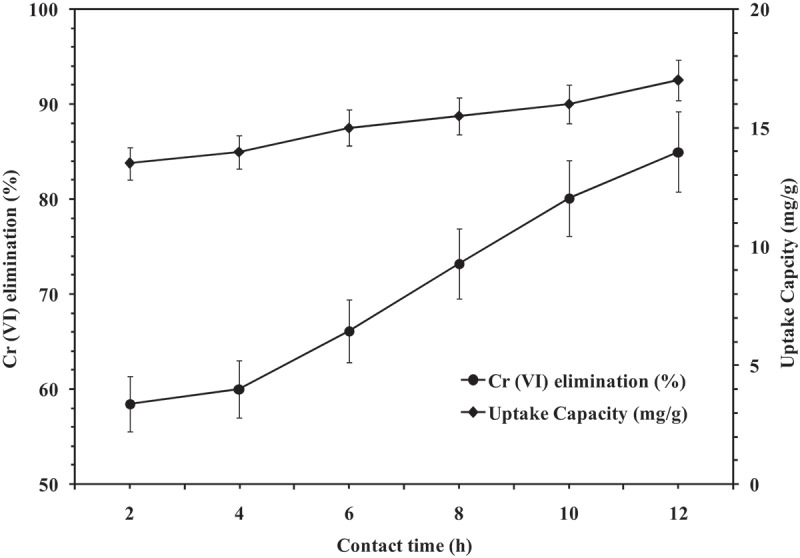
Influence of contact time on Cr (VI) elimination (Initial Cr (VI) concentration: 1000 ppm, Biosorbent dose: 20 g, pH: 2, Temperature: 37°C, Shaking speed: 120 rpm).

Cr (VI) elimination with respect to contact time is shown in Figure 5. This study was performed with synthetic effluent containing 1000 ppm Cr (VI) and 20 g/25 mL of biosorbent dose at pH 2.0 and 37°C. The optimum contact time for the maximum elimination of Cr (VI) was construed as 12 h. The percent uptake of Cr (VI) was increased from 58.44 ± 2.92% to 84.99 ± 4.25% during adsorption. Adsorption of the major portion of hexavalent chromium happened during the 12 h and it remained almost unchanged thereafter. Also, the removal of Cr (VI) occurred at a maximum extent during the earlier stage and eventually decreased and remained constant after the optimal time period [37]. Adsorption rate reduced in later periods because initially the number of vacant sites available on the surface for adsorption was large and as time passes the left out vacant sites were difficult to be occupied due to repulsive forces between the solute molecules of the solid and bulk liquid phase. Also, this result on comparison to a *Paecilomyces sp* based study for adsorption of Cr (VI) with a contact time of 7 days for 50 mg/L Cr (VI) may serve effective for the same purpose [27].

### 3.7. Adsorption isotherm

**Figure 6.**
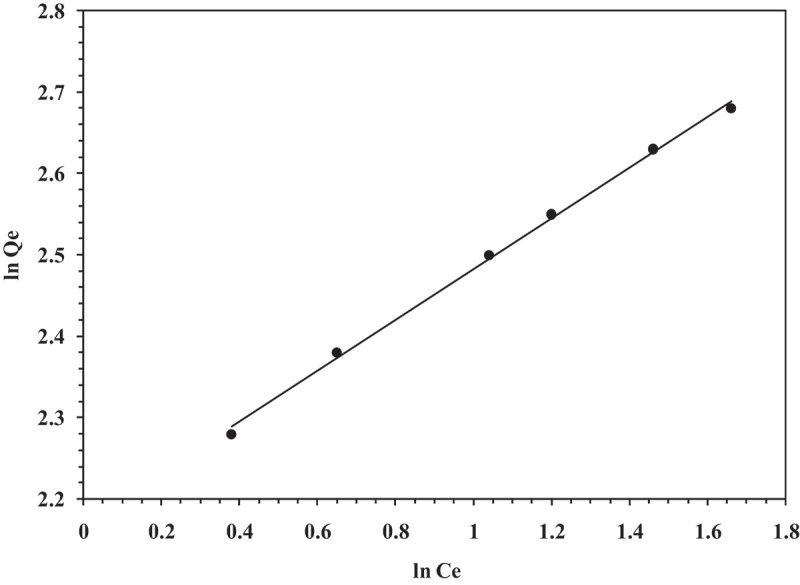
Freundlich Isotherm for Cr (VI) elimination (Initial Cr (VI) concentration: 1000 ppm, Biosorbent dose: 20 g/25 mL, pH: 2.0, Contact time: 12 h, Temperature: 37°C, Shaking speed: 120 rpm).

Based on the results obtained from biosorption in Cr (VI) solution, the change in biosorption by alginate-fungal biomass composites with initial metal concentration was elucidated corresponding to Langmuir and Freundlich isotherms. The isotherm constants have been determined to estimate extent of biosorption exhibited by the biosorbent. Biosorption kinetics for hexavalent chromium corresponded well with Freundlich isotherm model which is evident from a high regression coefficient (R^2^ = 0.99) (Figure 6). *K_f_* and *n* denote Freundlich biosorption isotherm constants indicating sorption efficiency of adsorbent and extent of biosorption correspondingly. A higher value of *K_f_* i.e. 0.90 suggests a high adsorption efficiency of the immobilized biomass. Furthermore, the value of n < 1 (0.86) indicates that the adsorption conditions are favorable.

### 3.8. Reusability of alginate-fungal biomass beads

Reusability of the biosorbent could decrease expenses of the heavy metal elimination processes currently being carried out [14]. The reusability of the polymer beads used has significant importance considering its large-scale use. Therefore, adsorption efficiency of regenerated alginate-fungal biomass composites in a solution containing hexavalent chromium was studied. Cr (VI) elimination rate by the alginate-fungal biomass beads was found to be 82.1 ± 4.1% after a successive cycle of biosorption and desorption post which desorption process was found to be insignificant due to rapid bead shrinkage. This may be attributed to the surface topological features and heterogeneous distribution of pores over the polymer bead. Also, in view of the contact time for a single cycle of adsorption which is almost 12 hours, the adsorption capacity of the beads showing 82.1 ± 4.1% removal after the second cycle was by itself intimidating. However, this could be improved by further optimizing the regeneration conditions.

## 4. Conclusions

The dead *A. carbonarius* biomass obtained from the remnants of enzyme and pigment production process had been utilized as a potential biosorbent for the elimination of hexavalent chromium ions in the prepared synthetic effluent. Under optimum conditions of 20 g/25 mL of biosorbent dosage at 37°C for 12 h with pH 2, alginate-fungal biomass composite exhibited 92.43 ± 4.02% of hexavalent chromium elimination. Further, all the reported experimental data had been appropriately analyzed using isotherm models and fitted with Freundlich isotherm model, thereby confirming its compatibility for biosorption. The study revealed that the alginate-fungal biomass composite has the potential to be utilized as an efficient biosorbent to sequester hexavalent chromium in an e-waste polluted environment due to its adsorption potential and cost-efficiency. Further, alginate-fungal biomass composite could be reused for subsequent studies.

## Highlights

- *Aspergillus carbonarius* biomass was used as a biosorbent for Cr(VI) elimination.
- Biosorption of Cr(VI) was fitted well with Freundlich Isotherm model.
- Reusability of alginate-fungal biomass bead eliminated ~82% Cr (VI) after a successive use.
- Removal of maximum of 92.43% hexavalent chromium was achieved.
